# How Can We Improve Patient Satisfaction As a Consumer of Public Health Services? The Case of Psychiatric Patients Undergoing Electroconvulsive Therapy

**DOI:** 10.3389/fpsyg.2016.00801

**Published:** 2016-05-26

**Authors:** Carmen Selva-Sevilla, Patricia Romero-Rodenas, Marta Lucas-Perez-Romero

**Affiliations:** ^1^Department of Applied Economics, University of Castilla-La ManchaAlbacete, Spain; ^2^Department of Psychiatry, University Hospital Complex of AlbaceteAlbacete, Spain

**Keywords:** mental disorders, electroconvulsive therapy/psychology, patient satisfaction, family/psychology, costs of health care

Electroconvulsive therapy (ECT) involves the induction of seizures for therapeutic purposes in cases of serious psychiatric disorders. Since the early twentieth century, ECT was one of the most important therapeutic alternatives to treat patients suffering from severe psychiatric disorders, and ECT maintained its therapeutic role despite the emergence of psychoactive drugs by mid-century. However, in the 1970's it fell into disuse due to the emergence of a psychiatric paradigm shift that considered it repressive and unsafe. The introduction of anesthesia as a fundamental part of the ECT has significantly improved the safety of the therapy, which, today, continues to be an effective therapy for its precise indications (Lopez Villaescusa et al., 2011; Rodriguez-Jimenez, 2015).

## Should we take into account the opinion of the psychiatric patient undergoing ECT?

The concept of “patient-centered care” consists of giving the patient an important role in making decisions about their health and, therefore, supporting the idea that clinical practice decisions should consider the patient's perspective. Consideration of the concept of patient satisfaction as an outcome of health care involves changes in the evaluation and improvement of the clinical practice (Mira and Aranaz, 2000).

For two decades, the so-called evidence-based medicine has emerged as a new paradigm in medicine. In this conception of medicine, the patient is considered to have an active part in the process of making decisions regarding their disease (Sackett et al., 1996). However, the real clinical application of the methods of evidence-based medicine has been distorted, especially with regard to the inclusion of patients in decision-making processes. There is currently a movement that calls for re-assessing the actual application that it is made of evidence-based medicine with the aim of returning to its original basis. In this sense, it is considered a key point the fact that patients will be taken into account in decision-making processes (Greenhalgh, 2014).

According with the proposal above mentioned, the mental health field has proposed that there is a need to evaluate the decision-making ability of psychiatric patients to ascertain if it is feasible to consider them in decision-making (Villagran et al., 2014). Even patients undergoing ECT have been considered for their degree of satisfaction with this therapy (Chakrabarti et al., 2010). In this sense, the success of healthcare would be related to patient satisfaction, perceived by them not only as the health outcomes achieved but also for the means used to achieve such outcomes (Mira and Aranaz, 2000).

In short, we believe that the outcome of healthcare should be measured in terms of not only effectiveness, safety, and efficiency but also the patient's perception of their autonomy and their sense of physical or mental well-being.

## What does the psychiatric patient undergoing ECT need in order to improve their satisfaction with the assistance received?

The inclusion of patient satisfaction as a criterion to develop clinical practice guidelines (Greenhalgh, 2014) is a highly recommended practice in the particular case of the psychiatric patient (Lasalvia and Ruggery, 2007; Khöler et al., 2015), and this approach is recommended because the rate of success of the chosen therapy may be much more dependent on psychosocial factors than in cases of non-psychiatric disorders.

The first step in learning the concerns of patients is asking for their opinion. Research about psychiatric patients' satisfaction usually involves the employment of questionnaires which are administered to patients and relatives. By doing so it has been identified that their perception about the health care was most influenced by four items: the competence of the care professionals who serve them, being given complete information about their diagnosis and treatment, being involved with their own treatment plan, and having the usefulness of their hospitalization explained to them (Berghofer et al., 2001; Blenkiron and Hammill, 2003; Gani et al., 2011; Fernández-Carbonell et al., 2012). ECT exhibits significant differential nuances, and therefore, specific questionnaires have been developed to ascertain the concerns of these patients, such as whether they face ECT with fear or if they believe that ECT is dangerous or painful, among others (Lopez Villaescusa et al., 2011; Rajagopal et al., 2013; Rodriguez-Jimenez, 2015).

## What should be done from the point of view of clinical practice to improve satisfaction of the patients undergoing ECT?

At present, ECT and psychotropic drugs coexist for the treatment of certain psychiatric disorders, but in some clinical conditions ECT is considered first-line treatment. Some of these indications arise from medical issues, such as when teratogenic psychotropic drugs must be avoided because of pregnancy, for instance, or when an outcome is required as rapidly as possible for patients suffering with severe life-threatening conditions. One of the main indications for ECT is, paradoxically, the patients' preference to receive ECT (Rodriguez-Jimenez, 2015). In addition, based on performance criteria, ECT has been more effective than drug treatment in some specific indications, such as severe depressive disorders (The UK ECT Review Group., 2003). This improved effectiveness affects the length of hospital stay for patients, which is shorter in duration (Markowitz et al., 1987) and which then leads to lower health care direct costs.

Considering the above information, our proposal for patients to undergo ECT earlier than is currently done seems feasible. To achieve this, a first step would be for physicians to *lose their fear* of ECT and consider applying it, in certain indications, without or before exhausting all of the possibilities of psychotropic drugs.

A second proposal would be to promote the use of ECT in continuation/maintenance cycles, which has been well appreciated by patients and physicians responsible for the therapy. ECT administered in continuation/maintenance cycles has been proven to have clinical efficacy and economic advantages by reducing the number of total days of hospitalization, the number of emergency room visits, and the number of emergency admissions for recurrences. In this regard, direct costs associated with the above mentioned concepts were lower after a continuation/maintenance program was implemented, as compared with the period before such program was implemented (American Psychiatric Association Committee on Electroconvulsive Therapy, 2001; Perestelo-Perez et al., 2013; Rodriguez-Jimenez et al., 2015).

A third proposal would be to act on those aspects related to ECT methodology by modifying both anesthetic and psychiatric protocols. Currently, there is a great disparity in the way in which ECT is performed between centers with regard to specific aspects, such as the employed hypnotic drug or the lower limit for an adequate electrical convulsion (Lihua et al., 2014). It is possible that the development of clinical guidelines based on the best available evidence and its widespread use afterwards would help to improve the quality of ECT sessions. This could then shorten the duration of the therapeutic process, which in turn would result in lower health care costs and, of course, in increased satisfaction of patients and relatives.

## Why is outpatient ECT not used?

The most effective way to shorten a hospital stay is by avoiding admittance. Currently, in most of the Mental Health Departments, patients remain hospitalized while undergoing all of the prescribed ECT sessions for the cycle of therapy. However, there is a real possibility to perform ECT in an outpatient way. These programs consist of admitting the patients to the hospital only for the duration of each ECT session, so that they remain at home for the duration of the prescribed ECT cycle (Kramer, 1990; Chan et al., 2006; Rodriguez-Jimenez, 2015). Of course, ECT outpatient programs should not be applicable for all psychiatric patients, but only to those patients meeting specific criteria, such as those that do not require continued psychiatric care and have good support from their relatives. These selected patients would benefit from the general advantages associated with any outpatient treatment, such as staying at home with their family for as long as possible; in our opinion, this course of action would yield clear psychological benefits for the psychiatric patients. It must be remembered that admission to a Unit of Acute Mental Disorders itself carries a burden of psychosocial stress that is linked to the feeling of a loss of freedom, which may be associated with an unfavorable outcome in this type of patient population.

In short, we believe that performing ECT in an outpatient way has two theoretical advantages: from a clinical point of view it can positively influence the way that the patients perceive their treatment process, and from an economic point of view it would reduce the costs derived from hospital stay.

## How would these actions that are aimed to improve clinical practice impact the satisfaction of psychiatric patients undergoing ECT?

In our opinion, it would improve their perception of the quality of received health care in three areas: clinical, psychological, and economic.

The clinical improvement would be because the quality of every single ECT session and the quality and efficacy of the therapy as a whole would be improved, as would the perception of the process by the patients.

The psychological improvement would be because improving the efficacy of clinical treatment and reducing the hospital stay would increase patients' comfort, as they could stay at home with their relatives, and would improve the psychological attitude of the patient toward ECT, as it would not be experienced as a negative element in their life.

The economic improvement would be because shortening the hospital stay would yield economic savings that could improve opportunity cost, and resources could be returned to the patients in other useful ways such as the acquisition of better ECT equipment and specialized medical staff.

Our final conclusion is that using patients' satisfaction as an indicator of the quality of health care processes should not be neglected when establishing programs to improve health care. In the particular case of psychiatric patients undergoing ECT, achieving the best possible patient satisfaction with the mental health care received is very important because it would have great influence on treatment adherence and, therefore, on clinical outcome.

## Author contributions

All authors listed, have made substantial, direct, and intellectual contribution to the work, and approved it for publication.

### Conflict of interest statement

The authors declare that the research was conducted in the absence of any commercial or financial relationships that could be construed as a potential conflict of interest.
